# Illumination of Parainfluenza Virus Infection and Transmission in Living Animals Reveals a Tissue-Specific Dichotomy

**DOI:** 10.1371/journal.ppat.1002134

**Published:** 2011-07-07

**Authors:** Crystal W. Burke, John N. Mason, Sherri L. Surman, Bart G. Jones, Emilie Dalloneau, Julia L. Hurwitz, Charles J. Russell

**Affiliations:** 1 Department of Infectious Diseases, St. Jude Children's Research Hospital, Memphis, Tennessee, United States of America; 2 Department of Microbiology, Immunology and Biochemistry, University of Tennessee Health Science Center, Memphis, Tennessee, United States of America; Erasmus Medical Center, Netherlands

## Abstract

The parainfluenza viruses (PIVs) are highly contagious respiratory paramyxoviruses and a leading cause of lower respiratory tract (LRT) disease. Since no vaccines or antivirals exist, non-pharmaceutical interventions are the only means of control for these pathogens. Here we used bioluminescence imaging to visualize the spatial and temporal progression of murine PIV1 (Sendai virus) infection in living mice after intranasal inoculation or exposure by contact. A non-attenuated luciferase reporter virus (rSeV-luc(M-F*)) that expressed high levels of luciferase yet was phenotypically similar to wild-type Sendai virus *in vitro* and *in vivo* was generated to allow visualization. After direct intranasal inoculation, we unexpectedly observed that the upper respiratory tract (URT) and trachea supported robust infection under conditions that result in little infection or pathology in the lungs including a low inoculum of virus, an attenuated virus, and strains of mice genetically resistant to lung infection. The high permissivity of the URT and trachea to infection resulted in 100% transmission to naïve contact recipients, even after low-dose (70 PFU) inoculation of genetically resistant BALB/c donor mice. The timing of transmission was consistent with the timing of high viral titers in the URT and trachea of donor animals but was independent of the levels of infection in the lungs of donors. The data therefore reveals a disconnect between transmissibility, which is associated with infection in the URT, and pathogenesis, which arises from infection in the lungs and the immune response. Natural infection after transmission was universally robust in the URT and trachea yet limited in the lungs, inducing protective immunity without weight loss even in genetically susceptible 129/SvJ mice. Overall, these results reveal a dichotomy between PIV infection in the URT and trachea versus the lungs and define a new model for studies of pathogenesis, development of live virus vaccines, and testing of antiviral therapies.

## Author Summary

Human parainfluenza viruses (HPIVs) are a leading cause of pediatric hospitalization for lower respiratory tract infection, yet it is unknown why primary infection typically induces immunity without causing severe pathology. To study the determinants of PIV spread within the respiratory tracts of living animals, we developed a model for non-invasive imaging of living mice infected with Sendai virus, the murine counterpart of HPIV1. This system allowed us to measure the temporal and spatial dynamics of paramyxovirus infection throughout the respiratory tracts of living animals after direct inoculation or transmission. We found that the upper respiratory tract and trachea were highly permissive to infection, even under conditions that limit lower respiratory infection and pathogenesis. The timing of transmission coincided with high virus growth in the upper respiratory tracts and trachea of donor mice independent of the extent of infection in the lungs. After transmission, infection spread preferentially in the upper respiratory tract and trachea, inducing protective immunity without weight loss. Our work reveals a disconnect between Sendai virus transmissibility and pathogenicity, and the experimental model developed here will be instrumental in studying PIV pathogenesis.

## Introduction

The parainfluenza viruses (PIVs) are non-segmented, negative-strand RNA viruses of the family *Paramyxoviridae*. The paramyxoviruses include not only the PIVs but also a number of other important human pathogens transmitted via the respiratory route such as human respiratory syncytial virus (HRSV), metapneumovirus, measles virus, and mumps virus [1], [2]. The human PIVs (HPIVs) consist of four serotypes (HPIV1-4), are a common cause of upper respiratory tract (URT) infections, and are a leading cause of lower respiratory tract (LRT) disease in infants and children [3]. The HPIVs are efficiently transmitted by direct contact and exposure to nasopharyngeal secretions [4], and nearly all children are infected with HPIV3 by age 2 and with HPIV1 and HPIV2 by age 5 [5], [6]. No licensed anti-PIV vaccines or drugs are available, and therefore non-pharmaceutical interventions are currently the only means of control. In view of these facts, an understanding of how PIV infection spreads within the respiratory tract, promotes pathogenesis, elicits immunity, and is transmitted to naïve hosts would greatly advance the development of novel vaccines and therapeutics.

Experimental studies of HPIV infection in tissue culture and animal models have helped reveal basic replication mechanisms and evaluate preclinical vaccine candidates [7]–[9]. However, knowledge about the spread of PIV infection in individual, living animals that are fully susceptible to PIV-associated disease would allow more thorough investigations of PIV virus-host interactions and transmission. Mice are poorly permissive to infection by the HPIVs, and HPIV infection in cotton rats, hamsters, guinea pigs, and ferrets is usually asymptomatic with minimal or undetectable pathology [1]. As a result, a number of studies have used Sendai virus (SeV) infection of mice as a model to investigate pathogenesis in an experimental setting [10], [11]. SeV is the murine counterpart of HPIV1, the leading cause of laryngotracheobronchitis (pediatric croup) [12]. SeV and HPIV1 have 78% amino-acid sequence identity [13], elicit cross-protective immunity [14]–[16], and have similar tissue tropism and epidemiology [1], [11]. Moreover, SeV shows promise as a Jennerian vaccine for HPIV1 [17] and as a vaccine vector for HRSV, HPIV3, and HPIV2 [18]–[20].

Although SeV and the HPIVs were first isolated in the 1950s and have been studied for more than 50 years [1], fundamental aspects of PIV infection and immunity that remain unknown are directly relevant to our understanding of pathogenesis and transmission. For example, the spatial and temporal spread of natural infection in the respiratory tract after SeV transmission remains poorly understood because of the ambiguous results (marked inter-animal variability and error) of classical experiments measuring virus titers in sacrificed mice [21], [22]. It is also unknown how HPIV and SeV infection after transmission often results in immunity without causing severe pathology. The contribution of LRT infection to transmission is unknown. Finally, while infection of the lungs and the concomitant immune response are clearly associated with disease severity [1], [11], [23], [24], many questions remain about the contribution of infection in the URT and trachea to clinical outcome and protective immunity [25], [26]. For example, we are unaware of any reports of studies investigating the effect of the dose of virus inoculum, the replicative fitness of the virus, or the genetic susceptibility of the host on the growth and clearance of SeV in the URT and trachea.

To measure the dynamics of PIV infection in living animals, we generated three luciferase-expressing SeVs that allow non-invasive *in vivo* bioluminescence imaging in mice. Analogous systems have previously been reported for DNA and positive-strand RNA viruses [27] but have been elusive for negative-strand RNA viruses, largely due to virus attenuation [28] or genetic instability resulting from reporter gene insertion [29]. We considered SeV an ideal candidate for non-invasive imaging because (i) foreign-gene expression by paramyxovirus vectors is usually genetically stable [30], (ii) *in vivo* imaging of a non-replicating SeV in intact mice has been successfully demonstrated [31] and (iii) the match of SeV and the murine host allows pathogenesis studies [11]. The reporter virus rSeV-luc(M-F*) described here was found to express high levels of luciferase yet replicate and promote disease in mice similar to wild-type (WT) virus. We imaged the dynamics of SeV infection in living, intact mice after direct inoculation and after contact transmission, varying both the virus dose and mouse strain. Unexpectedly, we observed a dichotomous tissue tropism in which the URT and trachea supported robust virus growth, efficient transmission, and protective immunity even under conditions that resulted in little infection in the lungs.

## Results

### *In vitro* properties of luciferase-expressing viruses

**Figure 1:**
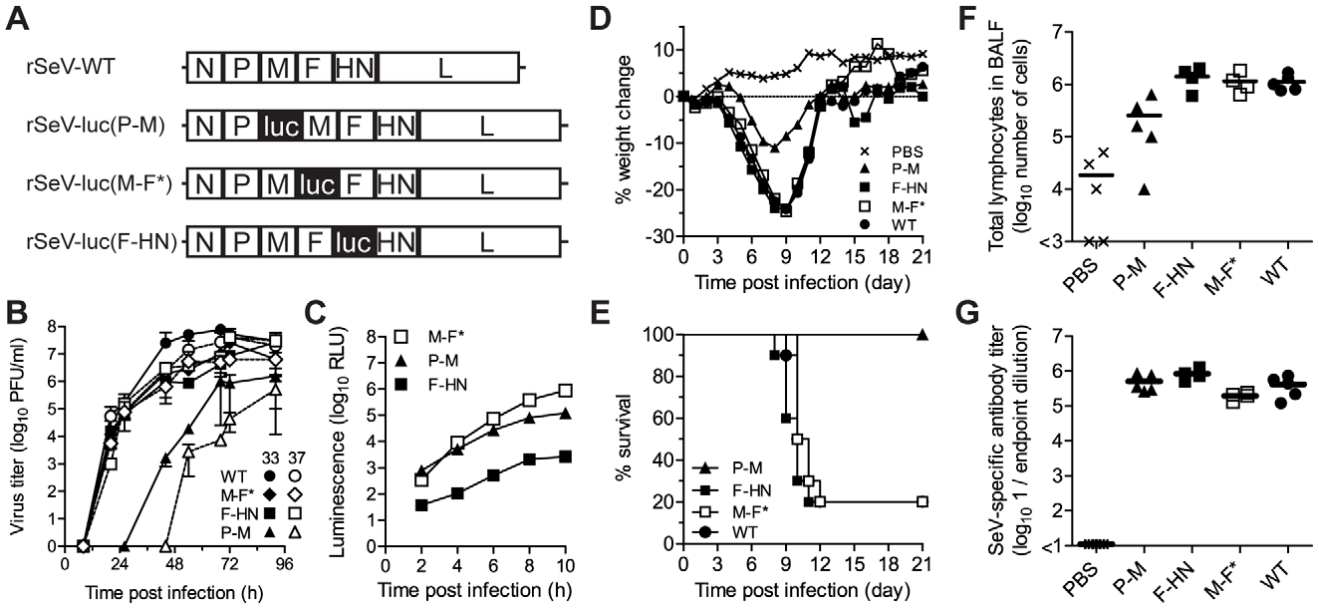
*In vitro* and *in vivo* phenotypes of luciferase-expressing Sendai viruses. (**A**) Recombinant Sendai viruses that contain the firefly luciferase gene (luc) inserted into the P-M, M-F, and F-HN gene junctions were generated. (**B**) Multiple-step replication kinetics of wild-type (WT) and luciferase-expressing Sendai viruses in LLC-MK2 cell cultures infected at a multiplicity of infection (MOI) of 0.01 PFU/cell and incubated at 33°C (closed symbols) and 37°C (open symbols). (**C**) Kinetics of luciferase reporter gene expression in LLC-MK2 cells infected with recombinant Sendai viruses at an MOI of 5 PFU/cell, as measured by luminescence. (**D**) Changes in mean body weight after intranasal inoculation of Sendai viruses. (**E**) Percent survival after intranasal inoculation of Sendai viruses. (**F**) Total lymphocyte counts in bronchoalveolar lavage fluid (BALF) 10 days after infection. (**G**) Sendai virus-specific binding antibody titers in sera collected 10 days after infection, as measured by reciprocal endpoint dilutions in ELISA assays. For panels **D**–**G**, groups of five 8-week-old 129/SvJ-strain mice were intranasally inoculated with 7,000 PFU of recombinant Sendai virus or phosphate buffered saline (PBS) and the experiments were performed twice. Cumulative data are shown in panels **D** and **E**, and representative data are shown in panels **F** and **G**.

To develop a model in which PIV infection could be visualized non-invasively in living, intact mice, we generated three recombinant Sendai viruses (rSeVs) in which a firefly luciferase reporter gene was inserted into the P-M, M-F, and F-HN gene junctions, respectively, of SeV (Figure 1A, Figure S1). Insertion of the firefly luciferase gene and gene junction into the SeV genome was expected to unacceptably decrease downstream viral gene expression and, consequently, virus replication [32]. To generate a luciferase-expressing SeV expected to suffer little or no attenuation, the rSeV-luc(M-F*) virus was constructed to contain both the luciferase reporter gene and a more efficient transcription start sequence AGGGTGAAAG upstream of the F gene (Figure S1). Therefore, the attenuating effects of reporter gene insertion could be counteracted by optimization of the naturally inefficient gene start sequence upstream of the F gene [33]. For the rSeV-luc(P-M) and rSeV-luc(F-HN) constructs, in which the luciferase gene was inserted into the P-M and F-HN gene junctions, respectively, the natural transcription start sequence upstream of the F gene was left intact (Figure S1).

Multiple-step growth curves were measured at 33°C and 37°C in LLC-MK2 cells that had been infected at a multiplicity of infection (MOI) of 0.01 PFU/cell (Figure 1B). Titers of rSeV-luc(M-F*) and rSeV-luc(F-HN) were similar at both temperatures and similar to SeV WT, showing that these two luciferase-expressing viruses were not substantially attenuated or temperature restricted. In contrast, the rSeV-luc(P-M) virus showed reduced growth kinetics at 33°C and grew even more slowly at 37°C. To compare luciferase reporter gene expression by the recombinant SeVs, we measured *in vitro* luciferase activity in LLC-MK2 cell lysates (MOI 5 PFU/cell) (Figure 1C). Upstream insertion of luciferase in rSeV-luc(P-M) resulted in greater luciferase activity than did downstream insertion in rSeV-luc(F-HN), consistent with the results of previous studies of SeVs using secreted alkaline phosphatase as the reporter gene [32]. Luciferase expression by rSeV-luc(M-F*) exceeded that of rSeV-luc(P-M) within 6 h post-infection (p.i.), showing that the enhanced gene start sequence engineered into the M-F* virus (Figure S1) increased reporter-gene transcription at later time points, perhaps due to greater downstream transcription of the L polymerase gene. To determine how the reporter gene insertions might have altered SeV protein expression, LLC-MK2 cells were infected at an MOI of 5 PFU/cell and lysates were subjected to radioimmunoprecipitation and SDS-PAGE analysis. Low levels of expression of the M, F, HN and presumably L proteins by the rSeV-luc(P-M) virus (Figure S2A) most likely contributed to the attenuation of this virus. Viral protein expression by rSeV-luc(M-F*) and rSeV-luc(F-HN) was sufficient to generate virions with WT-like compositions (Figure S2C), consistent with the *in vitro* growth of these two reporter viruses to levels similar to that of WT virus (Figure 1B).

### Virulence of the luciferase-expressing viruses

An ideal luciferase-reporter virus for non-invasive bioluminescence imaging and pathogenesis studies would express high levels of luciferase without altering virus replication and disease severity. To assess the virulence of the luciferase-expressing SeVs, 129/SvJ mice were anesthetized with isoflurane and inoculated intranasally with 30 µl containing 7,000 PFU of virus. This method of inoculation delivers ∼1/3 of the volume to the nasopharynx and ∼1/2 to the lungs [34]. Infection with SeV WT, rSeV-luc(M-F*), and rSeV-luc(F-HN) resulted in a mean weight loss of ∼25% and mean mortality rates of 80% (Figure 1D,E). Thus, these two luciferase-expressing viruses remained fully virulent at a dose of 7,000 PFU. In contrast, 129/SvJ mice inoculated with 7,000 PFU of the attenuated rSeV-luc(P-M) virus experienced only 12% weight loss and no mortality. All mice inoculated with 70,000 or 700,000 PFU of rSeV-luc(P-M) also survived (data not shown), further demonstrating that the attenuated rSeV-luc(P-M) virus is avirulent.

Acute viral pneumonia by SeV induces high levels of lymphocyte infiltration that show a peak in bronchoalveolar lavage fluid (BALF) at ∼10 d p.i. [35]. To compare lymphocyte influx caused by the luciferase-expressing viruses and WT virus, we sacrificed 129/SvJ mice that had been infected with 7,000 PFU at 10 d p.i. and collected BALF. Similarly large total numbers of lymphocytes, CD4+ T-lymphocytes, and CD8+ T-lymphocytes were detected in BALF after infection with WT, rSeV-luc(M-F*), or rSeV-luc(F-HN) virus (Figure 1F; Figure S3A–B), whereas mice inoculated with the attenuated rSeV-luc(P-M) had total lymphocyte counts only 10% as high. To determine the extents to which the reporter viruses elicited SeV- or luciferase-binding antibodies, ELISA assays were performed on sera collected at 10 d p.i. The anti-SeV antibody titers elicited by all three rSeVs were similar to that induced by WT virus (Figure 1G). The three reporter viruses also induced similar anti-luciferase antibody titers (Figure S3C). Thus, despite being attenuated and avirulent, rSeV-luc(P-M) elicited a robust antibody response. rSeV-luc(M-F*), which induced WT-like morbidity and mortality while expressing high levels of luciferase, was identified as the best suited surrogate for WT virus for use in subsequent bioluminescence imaging experiments.

### Dynamics of infection in living animals

In studies to determine whether non-invasive bioluminescence accurately reflected *in vivo* infection, 129/SvJ mice were intranasally inoculated with 7,000 PFU, imaged with a Xenogen IVIS instrument, and immediately euthanized. Respiratory tissues were promptly collected for *ex vivo* measurement of luminescence and viral titers. As in previous studies in immunocompetent mice [36], [37], viral titers and bioluminescence were limited to the respiratory tract. As shown in Figure S4, *in vivo* bioluminescence intensity levels in living animals were well correlated with *ex vivo* luminescence (*R* ^2^ 0.878) and with viral titers in the nasopharynx (*R* ^2^ 0.864), trachea (*R* ^2^ 0.915), and lungs (*R* ^2^ 0.961). The correspondence of these data validates the technique as a means of noninvasive measurement of infection *in vivo*. To determine whether the luciferase-reporter genes were genetically stable in the three rSeVs, we recovered lung tissues from 129/SvJ mice inoculated with 7,000 PFU of virus at 7 d p.i., homogenized the samples, and conducted plaque assays in LLC-MK2 cells. Five plaques of each of the three luciferase-expressing viruses were picked, amplified by one round of replication in eggs, RT-PCR transcribed, and sequenced. All of the individual viral clones contained the luciferase insert, which had no amino acid mutations, and expressed luciferase when grown in LLC-MK2 cells.

**Figure 2:**
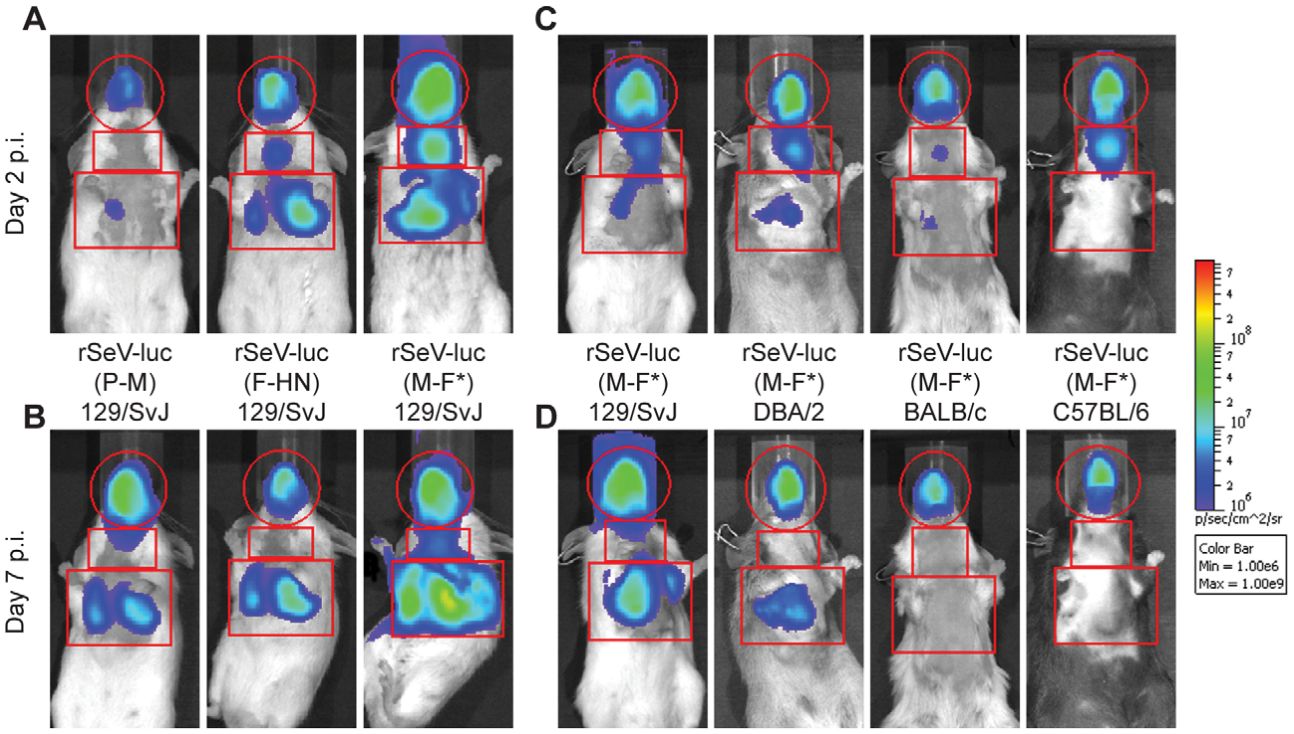
Non-invasive bioluminescence imaging of Sendai virus infection in the respiratory tracts of living mice. Eight-week-old mice were intranasally inoculated with 7,000 PFU of rSeV-luc(P-M), rSeV-luc(F-HN), or rSeV-luc(M-F*). Every 24 hours the mice were intraperitoneally injected with luciferin substrate, anesthetized with isoflurane, imaged with a Xenogen IVIS device, and then allowed to recover. Bioluminescence in one experiment is shown on day 2 (**A**) and day 7 (**B**) post-infection (p.i.) in 129/SvJ mice infected with rSeV-luc(P-M), rSeV-luc(F-HN), or rSeV-luc(M-F*). Bioluminescence in a second experiment is shown on day 2 (**C**) or day 7 (**D**) for 129/SvJ, DBA/2, BALB/c, or C57BL/6 mice infected with rSeV-luc(M-F*). The data are displayed as radiance (bioluminescence intensity) on a rainbow log scale with a range of 1×10^6^ (blue) to 1×10^9^ (red) photons/s/cm^2^/steradian. Red circles indicate regions of interest (ROI) used to calculate the total flux (photons/s) in the nasopharynx, and red rectangles indicate the ROI areas for the trachea and lungs.

**Figure 3:**
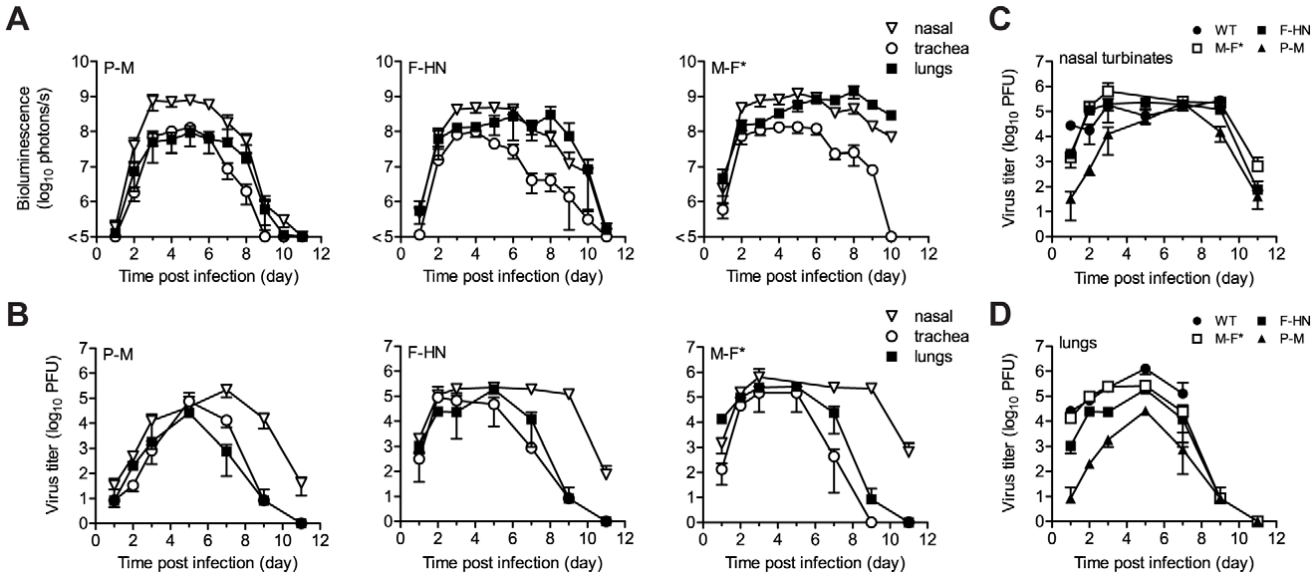
Kinetics of Sendai virus spread and clearance in the respiratory tracts of 129/SvJ mice. (**A**) The extent of infection was determined by non-invasive bioluminescence imaging of living, anesthetized mice every 24 h. Each data point represents the mean bioluminescence of 6 mice. The total flux (photons/s) of bioluminescence intensity is calculated as the sum of radiance in the region of interest. (**B–D**) Viral titers in the nasal turbinates, trachea, and lungs were determined by sacrificing groups of 3 mice at the reported days and performing plaque titrations of tissue homogenates in LLC-MK2 cells. Both experiments were repeated, and representative data are shown.

We next measured the kinetics and tropism of bioluminescence in living 129/SvJ mice and compared the results to conventionally measured viral titers in dissected tissues (Figures 2 and 3). Just as rSeV-luc(M-F*) and rSeV-luc(F-HN) had *in vitro* replication rates and *in vivo* pathogenicity similar to those of WT virus, they also had WT-like titers in the nasal turbinates, trachea, and lungs. In the nasal turbinates, high virus titers (>10^5^ PFU) were detected by day 2 p.i. and were maintained until day 9 p.i., after which rapid clearance occurred (Figure 3B). Between days 2 and 9 p.i., high levels of *in vivo* bioluminescence were similarly observed in the nasopharynx (>10^8^ photons/s) of 129/SvJ mice infected with rSeV-luc(M-F); bioluminescence peaked at about 5 d p.i. (Figure 3A). In the lungs, the titers of all three luciferase-expressing viruses and of WT SeV peaked by day 5 p.i. and fell to low levels by day 9 p.i. Infection with the attenuated rSeV-luc(P-M) resulted in peak lung titers of ∼10^4^ PFU (approximately 5% of the WT titer) at day 5 p.i. (Figure 3D). Similarly low levels of rSeV-luc(P-M) bioluminescence were observed in the lungs (Figure 3A), consistent with the attenuated and avirulent virus phenotype. On the other hand, rSeV-luc(P-M) reached high peak titers (∼10^5^ PFU, similar to the WT titer) in the nasal turbinates at 7 d p.i. (Figure 3C), and high levels of bioluminescence were observed in the nasopharynx between days 3 and 7 p.i. (Figure 3A).

### Tissue tropism and viral dose

**Figure 4:**
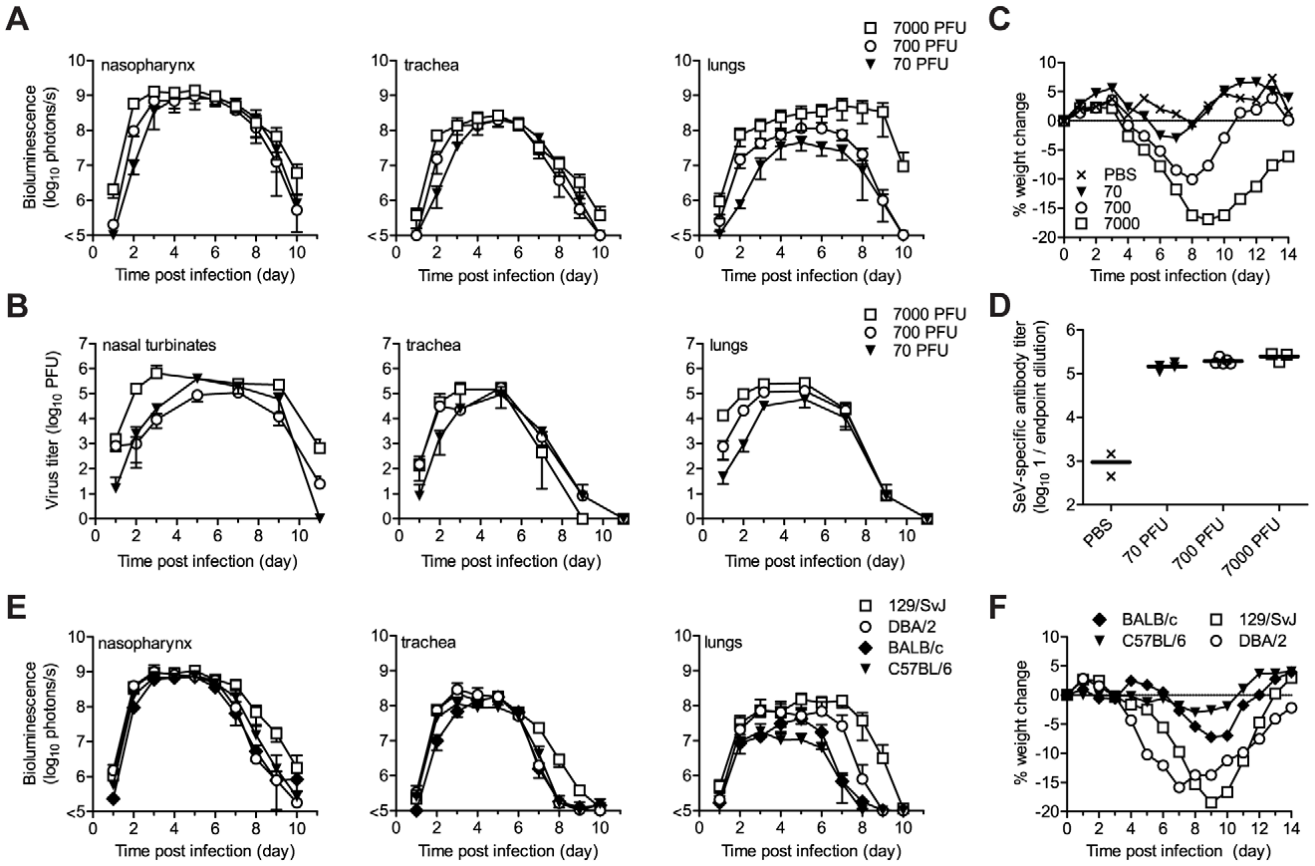
Virus replication and pathogenesis as a function of virus dose and mouse strain. After intranasal inoculation of 129/SvJ mice with 70 to 7,000 PFU of rSeV-luc(M-F*), the total flux of bioluminescence intensity (**A**) and viral titers (**B**) were measured as described in Figure 3. (**C**) Percent body weight change was measured in groups of ten 129/SvJ mice after inoculation with 70 to 7,000 PFU. The experiment was performed in duplicate; representative data are shown. (**D**) Sendai virus-specific binding antibody titers in sera of 129/SvJ mice collected 10 days after inoculation with 70 to 7,000 PFU of rSeV-luc(M-F*). Titers are reported as the reciprocal endpoint dilutions in ELISA assays. Five infected and two control mice were used in the experiment, which was performed in duplicate. Representative data are shown. (**E**) The total flux of bioluminescence intensity in the nasopharynx, trachea, and lungs after 7,000-PFU intranasal inoculation of 129/SvJ, DBA/2, BALB/c, or C57BL/6-strain mice with rSeV-luc(M-F*). Values are the mean from six animals. The experiment was performed in duplicate, and the results from a representative experiment are shown. (**F**) Mean percent weight change in groups of 10 mice after infection with 7,000 PFU of rSeV-luc(M-F*). The experiment was performed in duplicate; representative data are shown.

The high permissivity of the URT and trachea to infection by the attenuated rSeV-luc(P-M) virus was unexpected. We next investigated whether these tissues were also highly permissive to infection by the WT-like virus rSeV-luc(M-F*) at a low inoculating dose. Our preliminary studies showed that the 50% mouse infectious dose (MID_50_) of rSeV-luc(M-F*) was 9 PFU and that a 70-PFU dose resulted in 100% infection, similar to results obtained for WT SeV in mice [38] and HPIV1 in humans [39]. We inoculated 129/SvJ mice intranasally with 70, 700, or 7,000 PFU of rSeV-luc(M-F*) in equal 30 µl volumes and then measured bioluminescence and viral titers. After inoculation with 70 PFU, viral titers and bioluminescence in the lungs were ∼10% of that induced by a 7,000-PFU dose (Figure 4A,B), and weight loss was far less (Figure 4C). In contrast, infection in the nasopharynx and trachea after a 70-PFU inoculation was delayed ∼1 d compared to 7,000-PFU inoculation, reached a similar level by ∼5 d p.i. (Figure 4A,B), and induced relatively high titers of SeV-specific antibodies (>10^5^) (Figure 4D). Therefore, low-dose inoculation of the WT-like rSeV-luc(M-F*) virus resulted in preferential infection of the URT and trachea, inducing a robust antibody response without causing much weight loss.

### Tissue tropism and host genetics

While it is known that 129/SvJ and DBA/2 mice are highly susceptible to lung infection by SeV and BALB/c and C57BL/6 mice are highly resistant [40]–[43], the effect of host genetics on SeV replication in the URT and trachea has not been reported. Therefore, we measured the *in vivo* dynamics of SeV infection in 129/SvJ, DBA/2, C57BL/6, and BALB/c strains of mice that had been intranasally inoculated with 7,000 PFU of rSeV-luc(M-F*). As expected from previous studies, the extent of pulmonary infection and weight loss correlated with each other and followed the trend C57BL/6<BALB/c<<DBA/2<129/SvJ (Figure 4). In contrast, similarly high levels of bioluminescence were observed in the URT and trachea in all four strains of mice. The titers of rSeV-luc(M-F*) in BALB/c mice correlated with bioluminescence in intact mice (Figure S5A), as they were in 129/SvJ mice. Therefore, use of the bioluminescence technique to measure respiratory tract infection in living mice was validated in both 129/SvJ and BALB/c strains.

### Dynamics of infection during contact transmission

**Figure 5:**
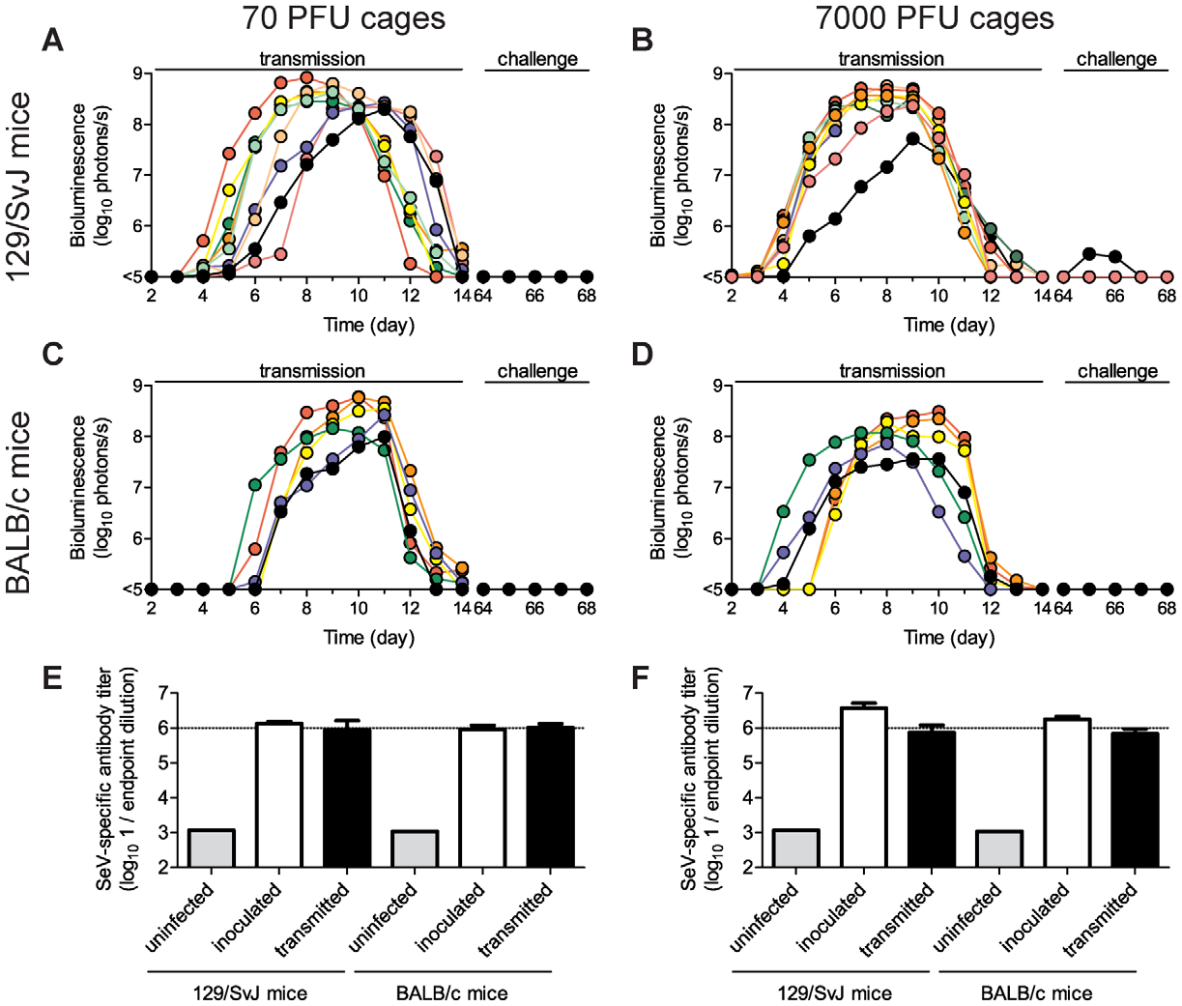
Sendai virus infection and immune response after contact transmission. Donor mice were directly inoculated with 70 PFU (**A,C,E**) or 7,000 PFU (**B,D,F**) of rSeV-luc(M-F*) and then introduced into a cage with 3 naïve animals one day later. The total flux of bioluminescence intensity in the nasopharyngeal cavities of individual 129/SvJ (**A–B**) and BALB/c (**C–D**) mice are shown. Serum was collected on day 60 and contact mice were challenged with 7,000 PFU of rSeV-luc(M-F*) on day 63 to monitor for re-infection by bioluminescence. Sendai virus-specific binding antibody titers were measured as reciprocal endpoint dilutions of sera collected on day 60 from mice co-housed with animals inoculated with 70 PFU (**E**) or 7,000 PFU (**F**). Open bars represent mice inoculated on day 0 and solid bars represent the contact mice. The experiment was performed in triplicate for 129/SvJ mice (3 donor and 9 contact mice) and duplicate for BALB/c mice (2 donor and 6 contact mice).

**Figure 6:**
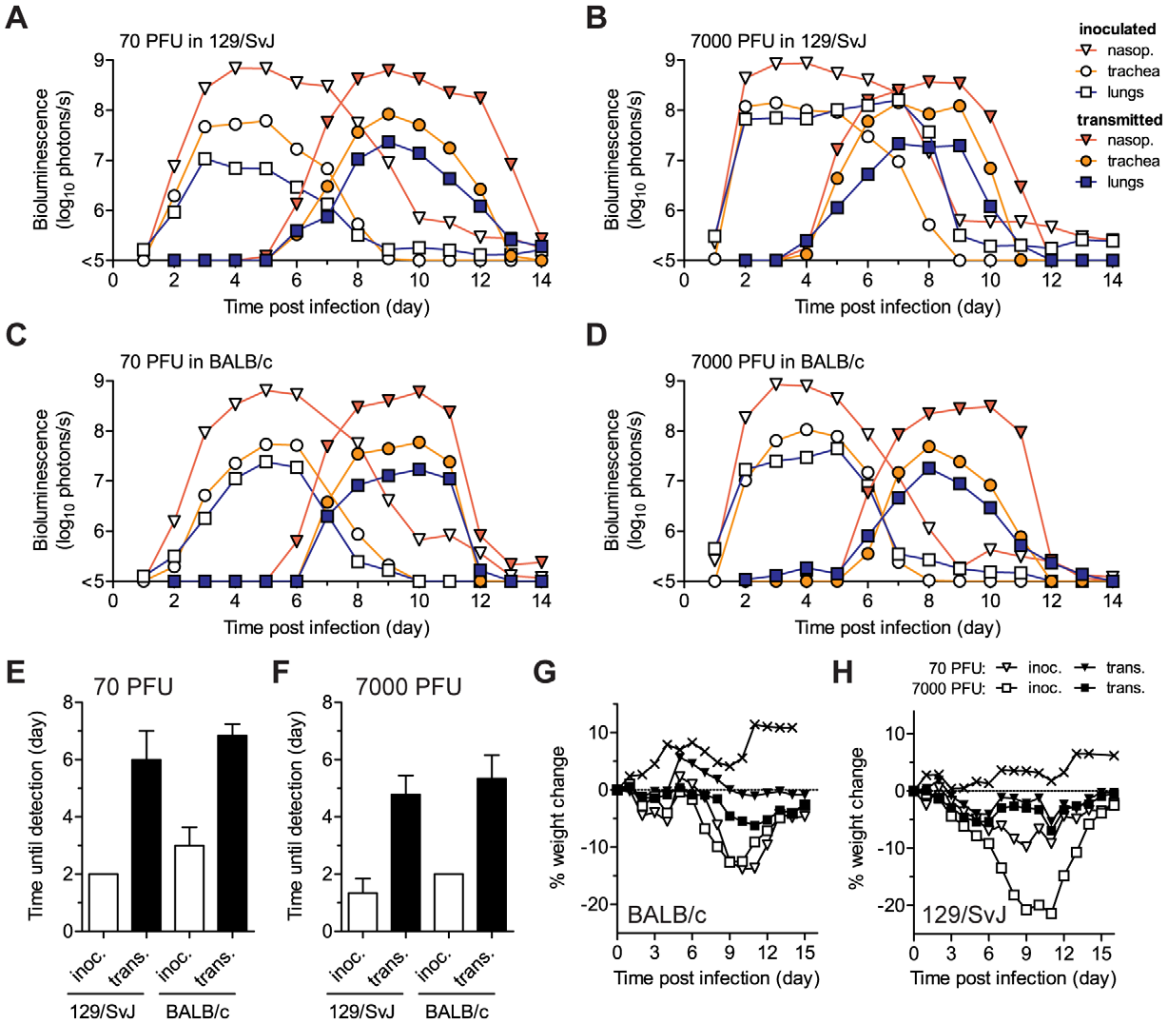
Timing and tissue-tropic spread of Sendai virus infection after contact transmission. The co-housing of contact mice with mice inoculated with rSeV-luc(M-F*) is described in Figure 5. The total flux of bioluminescence intensity in individual, representative 129/SvJ (**A–B**) and BALB/c (**C–D**) mice is shown for the nasopharynx (triangles), trachea (circles), and lungs (squares). Time to detection of bioluminescence (>6 log_10_ photons/s) in the nasopharynx after inoculation of donors with either 70 PFU (**E**) or 7,000 PFU (**F**) of virus. Mean percent weight change in BALB/c (**G**) and 129/SvJ (**H**) mice. The contact transmission experiment was performed in triplicate for 129/SvJ mice and in duplicate for BALB/c mice. Open symbols and bars represent directly inoculated mice and solid symbols and bars represent contact mice. In panels **G** and **H**, the symbol X indicates PBS-inoculated control mice.

SeV, the HPIVs, and HRSV are thought to be transmitted primarily via contact with respiratory secretions as opposed to long-range transmission of these secretions as small-particle aerosols [21], [22], [24], [44], [45]. It has also been shown that growth of SeV [21] and influenza virus [46] in the URT promotes transmission. Two fundamental questions about PIV transmission that have long remained unanswered are (i) how growth of virus in the lungs of donors influences transmission and (ii) how infection spreads in the respiratory tracts of contact animals after transmission. To address these fundamental questions about SeV transmission, we inoculated BALB/c or 129/SvJ donor mice with 70 or 7,000 PFU of rSeV-luc(M-F*) and then at 1 d p.i. we placed 1 donor mouse in a “clean” cage with 3 naïve contact mice. We measured bioluminescence daily in inoculated and contact mice until primary infection was cleared. We then collected sera on day 60, challenged the mice with 7,000 PFU of rSeV-luc(M-F*) on day 63, and subsequently imaged the mice daily to detect reinfection (Figures 5 and 6). Transmission to every naïve contact mouse was observed by nasopharyngeal bioluminescence and seroconversion, including resistant BALB/c mice exposed to donor animals inoculated at the lower dose of 70 PFU (Figure 5). The timing of transmission was not influenced by the extent of lung infection in donors, as lung titers were ∼10 times lower in BALB/c than in 129/SvJ donor mice after 7,000-PFU inoculation (Figure S5C), yet the timing of transmission (time between detection in inoculated animals and contact animals) was similar (3.3 and 3.4 days, respectively) (Figure 6F). While LRT infection occurred in both strains of mice and may have contributed to transmission, the primary determinant of transmission appeared to be virus shedding in the URT and trachea. For example, both high-titer (>10^5^ PFU) shedding in the nasal cavities and trachea of 129/SvJ donor mice (Figure 4A,B) and contact transmission (Figure 6E,F) occurred ∼1 day earlier after 7,000-PFU inoculation than after 70-PFU inoculation.

Under all four conditions tested (129/SvJ or BALB/c donor mice infected with 70 or 7,000 PFU of virus), the tropism and magnitude of infection in contact animals was similar to that observed after direct intranasal inoculation with 70 PFU of rSeV-luc(M-F*). After contact transmission, bioluminescence was first observed in the nasopharynx and then spread to the trachea and lungs an average of 0.8 and 1.0 days later, respectively (Figure S6A–D). Robust infection was observed in the nasopharynx and trachea after transmission (Figure 6A–D, Figure S6E–H). In contrast, low levels of infection in the lungs were observed after transmission, consistent with low weight loss (Figure 6G–H). In all four groups of mice, SeV-specific antibody titers on day 60 were similarly high (∼10^6^) and all animals were protected from challenge on day 63 (Figure 5). After challenge, a low level of bioluminescence (<10^6^ photons/s), but no weight loss, was detected in only 1 of the 30 contact mice; this animal had shown the lowest level of bioluminescence on days 5–12 after primary infection (Figure 5B, solid black circles). As this animal also had the lowest level of SeV-specific antibodies at day 60 before challenge, a threshold level of infection may be required to induce the highest levels of protective immunity. Overall, SeV infection after transmission was observed to be sufficiently robust in the URT and trachea, yet sufficiently limited in the lungs, to induce protective immunity without causing severe pathogenesis.

## Discussion

In this study, we generated and used luciferase-reporter viruses to study the kinetics of SeV infection in living mice after direct inoculation or contact transmission. WT SeV virus and the luciferase-expressing virus rSeV-luc(M-F*) had a similar replication rate *in vivo* and elicited similar levels of weight loss, mortality, bronchoalveolar lymphocyte influx, and serum antibody titers. Both susceptible (129/SvJ) and resistant (BALB/c) strains of mice were intranasally inoculated with 70- and 7,000-PFU doses of rSeV-luc(M-F*), and the spread of infection was measured by both *in vivo* bioluminescence in intact mice and *ex vivo* virus titers in the tissues of sacrificed animals. The consequences of infection in the URT and trachea were found to be distinct from those of infection in the lungs. Unexpectedly, under all conditions tested, including 70-PFU inoculation of resistant BALB/c mice, the URT and trachea supported robust SeV growth, efficient contact transmission, and protective immunity independently of the extent of infection in the lungs. In contrast, the extent of infection in the lungs varied with the virus dose and mouse strain and was highly correlated with weight loss and mortality. Overall, the results reported here reveal a tissue-specific dichotomy in the respiratory tract in which robust infection in the URT and trachea supports efficient transmission while the extent of infection in the lungs and the host response determines disease severity.

Here we describe for the first time the development of a non-invasive bioluminescence imaging system to visualize negative-strand RNA virus infection throughout the bodies of living animals, using the respiratory paramyxovirus SeV as a model. The development of a non-attenuated paramyxovirus that expresses sufficiently high levels of a reporter gene to allow non-invasive imaging of small animals has been challenging because of the polarized transcription mechanism of these non-segmented negative-strand RNA viruses [2]. A significant advance described here is the generation of the rSeV-luc(M-F*) virus, in which the attenuating effect of reporter-gene insertion [32] is counteracted by enhancement of the naturally occurring but suboptimal start sequence upstream of the F gene [33]. Expression of the F gene, a virulence factor [47], [48], is also downregulated by HPIV1 [49], HPIV3 [50], PIV5 [51], measles virus [52] and canine distemper virus (CDV) [47] by readthrough transcription or long untranslated regions. Therefore, we predict that other WT-like reporter paramyxoviruses that express high levels of luciferase can be engineered by inserting the reporter gene into the M-F junction and maintaining F gene expression through compensating mutations. Reporter gene expression without attenuation of SeV has also been achieved by construction of a bicistronic gene that contains an internal ribosome entry site [53], although it is not yet clear whether this approach yields sufficient luciferase expression to allow non-invasive imaging of *in vivo* infection.

The use of the luciferase reporter gene in the present work enabled the measurement of infection throughout the entire respiratory tracts of intact animals, allowing us to measure the spread and clearance of infection after direct inoculation or transmission. eGFP-expressing reporter viruses have also been used to study the dynamics of CDV infection in ferrets [54], [55] and measles virus infection in monkeys [56], [57]. The eGFP reporter gene provides the advantage of allowing the tropism of infection to be studied at the cellular level in dissected tissues. Moreover, eGFP-expressing viruses can also be used to quantify and type infected cells in the peripheral blood, skin, and mouths of living animals. eGFP-expressing HPIV3 and Sendai viruses have been used to study the cellular tropism of PIV infection in well differentiated, primary epithelial cultures. In the case of HPIV3, infection was found to be restricted to ciliated epithelial cells and to cause little cytopathology [58]. In contrast, SeV was found to infect ciliated and non-ciliated cells, but not goblet cells, and was observed to induce ciliostasis, cell sloughing, apoptosis, and cellular degeneration [59]. It is unknown whether cell-free virus or cell-associated virus is associated with SeV transmission.

A major finding reported here is that the efficiency and timing of SeV transmission are independent of the extent of pulmonary infection, clinical symptoms, and host genetics. HPIV1 transmission from asymptomatic human donors has also been observed in an experimental setting [39] and is consistent with epidemiological observations for PIV outbreaks in general [23], [24]. These observations suggest that LRT infection and the severity of clinical symptoms are poor predictors of transmission potential in surveillance and infection control efforts. As in previous work [21], [38], we observed that SeV transmission coincides with high-titer virus growth in the URT and is remarkably efficient because of the high infectivity of the virus (e.g., the MID_50_ of SeV is <10 PFU). HPIV1, HPIV3, and HRSV are similarly highly infectious and also transmit predominantly by direct contact or indirect exposure to nasal secretions [44], [45], [60]–[62]. In the absence of an available prophylactic drug for uninfected individuals in high-risk groups (e.g., premature infants and the immunocompromised), the results described here suggest that efforts to control PIV infection should focus on reducing URT shedding from infected individuals, disinfecting contaminated surfaces, and hand washing. In contrast to infection control, which would be best served by limiting URT infection, therapeutic antivirals would be better targeted to the LRT to control clinical manifestations of PIV-associated disease.

Genetic factors have been identified that modulate viral susceptibility and disease severity in humans [63]–[65] and in the lungs of mice [40], [42], [43], [66]–[70]. Our results show for the first time that genetic factors limiting virus growth in the lungs of resistant BALB/c mice, compared to susceptible 129/SvJ mice, do not limit robust virus growth in the URT and trachea and, consequently, do not limit transmission. Furthermore, BALB/c and 129/SvJ mice showed a similarly high extent of infection in the URT and trachea and a similarly low extent of infection in the lungs after exposure to cagemates inoculated with high or low virus doses. This finding shows that host genetics do not play a major role in SeV transmission, at least in these strains of mice. These observations reinforce our inference that transmission and pathogenesis are independent consequences of URT versus LRT infection, respectively. Additional experiments are needed to delineate the mechanisms responsible for the higher permissivity of the URT and trachea than of the lungs to SeV infection. Possible mechanisms include the site of inoculation in the nasal cavity, lower temperature in the URT, tissue-specific differences in virus replication and innate immunity, and antiviral mechanisms, such as surfactant proteins in the lungs. Reduced replication in the lungs may be associated with lower levels of the secreted tryptase Clara, which is required for cleavage of the F protein to allow viral entry [71], [72].

Asymptomatic infection that promotes immunity and transmission represents a balanced relationship that benefits both the virus and the host. Such has been the case in several enzootic (clinically unapparent) epidemics of SeV in which subclinical infections were maintained in mouse and hamster colonies for years with no increase in pathogenicity, causing apparent disease only occasionally in suckling and old animals [73], [74]. These epidemiological observations are reminiscent of the low virulence yet high transmissibility of the reverse-genetics engineered SeV described here, which was derived from a modified Enders strain that had been passaged in embryonated chicken eggs. Our results show that the level of virus replication in the lungs affects neither the timing nor the efficiency of transmission; thus, SeV replication in the lungs may offer no selective advantage. Instead, we suggest the following mechanism for symbiotic virus-host interplay in enzootic epidemics of SeV: natural infection after transmission is sufficiently limited in the lungs to avoid clinical signs of disease yet is sufficiently robust in the nasopharynx and trachea to promote efficient transmission and induce protective immunity.

Epizootic (clinically apparent) outbreaks of SeV have caused morbidity and high rates of mortality in mouse colonies [75]–[77]. Two closely related, highly pathogenic field isolates of SeV are the Ohita and Hamamatsu strains [78], [79]. While inoculation with only a few PFU of unpassaged Hamamatsu strain SeV results in mortality in mice, the MLD_50_ of the virus was attenuated by as much as 400-fold after 50 passages in eggs [80]. When the highly pathogenic Ohita and Hamamatsu strains were adapted to LLC-MK2 cells and chicken eggs, they were found to have selected for mutations in the C protein and untranslated leader region, respectively, which increase replication in cultured cells but attenuate replication and pathogenesis in the lungs of mice [81]–[83]. The bioluminescence imaging system described here would be useful in determining whether the mutations that attenuate replication in the lungs also attenuate replication in the URT and trachea, thereby reducing transmission, or whether they actually promote sustained transmission by supporting nasal and tracheal shedding of virus while reducing pathogenesis in the lungs. Such experiments may also reveal whether our observations about the spread and transmission of the egg-adapted SeV extend to unpassaged, highly pathogenic field isolates.

SeV is a promising Jennerian vaccine against HPIV1 [1], [13], and recombinant SeV vaccine vectors containing an envelope gene from HRSV, HPIV3, or HPIV2 inserted into the F-HN gene junction have been shown to elicit both B- and T-cell responses that lead to protection from challenge in small-animal models [18]–[20]. While SeV is pathogenic in mice, an ongoing clinical trial has demonstrated SeV to be well tolerated in humans [17]. In non-human primates, SeV has been shown to protect against HPIV1 challenge with no associated adverse events [15], [84]. This result is likely due in part to the sensitivity of SeV to human IFN-mediated innate immunity [85]. As SeV is developed further as a vaccine vector, the luciferase-expressing SeVs, imaging system, and methods described here will be useful in investigating the effect of vaccine dose, volume, and position of foreign antigen insertion in the SeV genome on tissue-specific vector growth and the immune response in small animal models. Of course, replacing the luciferase reporter gene in SeV with a vaccine antigen could alter *in vivo* replication of the vector. For example, three different recombinant HPIV3 vectors expressing HPIV1 HN, HPIV2 HN, or measles virus HA inserted into the P-M gene junction were found to replicate to different levels in hamsters [86].

In summary, we have described the development of the non-attenuated reporter virus rSeV-luc(M-F*), which can be used to quantify tissue-specific SeV infection in living mice. Our results reveal how infection by SeV spreads in individual, living animals after direct intranasal inoculation and after transmission. Importantly, infection in the URT and trachea were found to be associated with contact transmission while infection in the lungs was found to be associated with pathogenesis. The imaging tools developed here will provide a method to study the effect of viral factors, host genetics, host age, immune status, environmental conditions, and inoculation mode on the dynamics of infection and transmission. For example, infection can be tracked non-invasively in WT and knockout mice before immune responses are measured *ex vivo* and then interpreted in light of the preceding infection. Methods similar to those reported here could also be developed to image infection by other paramyxoviruses in small-animal models. Overall, our model system and results suggest tissue-targeted approaches to PIV infection control and vaccine development, while our non-invasive bioluminescence imaging technique is expected to advance the preclinical testing of candidate vaccine vectors and experimental therapies.

## Materials and Methods

### Ethics statement

All animal studies were approved by the Animal Care and Use Committee of St. Jude Children's Research Hospital and were performed in compliance with relevant institutional policies, the Association for the Accreditation of Laboratory Animal Care guidelines, the National Institutes of Health regulations, and local, state, and federal laws.

### Cell culture

Monolayer cultures of LLC-MK2 cells were grown in Dulbecco's minimal essential medium (DMEM) supplemented with 10% fetal bovine serum, 1% L-glutamine, 1% penicillin, and 1% streptomycin at 37°C, 5% CO_2_.

### Recombinant Sendai viruses

Unique *Not*I recognition sites were cloned into the P-M, M-F, and F-HN intergenic junctions of an Enders-based pSeV viral genome plasmid using cloning sites described previously [32]. The firefly luciferase gene was amplified by PCR using the pGL3 Basic vector (Promega) and a pair of *Asc*I tagged primers, subcloned into a shuttle plasmid containing a SeV intergenic junction and flanking *Not*I restriction sites [32], and then subcloned into the unique *Not*I site of each of the pSeV viral genome plasmids. Within the pSeV-luc(M-F*) plasmid, the start signal upstream of the F protein was changed from AGGGATAAAG to AGGGTGAAAG by using the QuikChange Site-Directed Mutagenesis Kit (Stratagene Corp). The rSeVs were rescued from the pSeV genome plasmids as described previously [20].

### Luciferase expression *in vitro*

rSeV-infected LLC-MK2 cells (MOI, 5 PFU/cell) were incubated at 33°C, 5% CO_2_, and lysates were collected at various times p.i.. Luciferase assays were performed using the Luciferase Assay System (Promega) and expression was measured on an automated luminometer (Turner Biosystems, Inc.) as described previously [87].

### Viral titers and bioluminescence imaging

Virus titers from multistep growth curves (MOI of 0.01 PFU/cell) and homogenized tissues were determined by plaque titration in LLC-MK2 cells as described previously [48]. Eight week-old female 129/SvJ mice or BALB/c mice (Jackson Laboratories) were anesthetized with isoflurane (Baxter Health Care Corp.) and inoculated intranasally (i.n.) with 30 µl of PBS or PBS containing virus. Animals were monitored daily for weight loss, morbidity, and mortality. Before imaging, mice were injected intraperitoneally with luciferin (Xenogen Corp) at a dose of 150 mg/kg of body weight and anesthetized with isoflurane for 5 min. *In vivo* images were acquired with a Xenogen IVIS CCD camera system (Caliper Life Sciences) and analyzed with Living Image 3.2 software (Caliper Life Sciences) using an exposure of 60s, 30s, or 5s (binning 4; f/stop 1). Pseudocolor images (representative of bioluminescence) of mice were displayed using a binning of 4 on a colorimetric scale ranging from 1×10^6^ to 1×10^9^ surface radiance (photons/s/cm^2^/steradian), which is defined as the number of photons that leave a cm^2^ of tissue and radiate into a solid angle of one steradian. To quantify bioluminescence, regions of interest (ROI) were defined manually and graphed data were expressed as total flux (photons/s), which is defined as the radiance within each pixel summed over the ROI area (cm^2^)×4π. For experiments shown in Figures 1D, 1E, 2, 3, 4E, 4F, and S4, mice were anesthetized by IP injection of 300 µl 2,2,2-Tribromoethanol (300 mg/kg) and chest hair was removed by shaving and application of a depilatory cream 3 d before inoculation.

### Immunology

Sera and BALF were collected from euthanized animals on day 10 or day 60 p.i.. BALF samples (3 ml) were centrifuged to collect cellular material and plated in a tissue culture dish for 1 h at 37°C to remove adherent cells. Suspension cells were harvested, total lymphocytes were counted microscopically, and red blood cells were lysed. For analyses by flow cytometry, cells were stained with FITC-conjugated anti-CD4 (RM4-4) and PE-conjugated anti–CD8b (53-5.8) antibodies (BD Biosciences Pharmingen). Lymphocytes were gated based on forward and side scatter, and the percentages of CD4+ and CD8+ T cell populations were measured within this gate. ELISAs were used to measure the levels of SeV-specific or luciferase-specific antibodies present in the sera. Briefly, 96-well plates were coated overnight with disrupted, purified SeV (10 µg/ml) or firefly luciferase (1 µg/ml, Abcam). Plates were blocked with PBS containing 1% BSA and then incubated with 10-fold serially diluted serum samples. After incubation, plates were washed, incubated with HRP-Goat anti mouse IgG (Southern Biotechnologies) and then washed further. To quantify levels of antibodies, TMB substrate (Kirkegaard and Perry Laboratories) was added to the wells followed by stop solution and absorbance was read at a wavelength of 450 nm. GraphPad Prism non-linear regression software was used to calculate antibody titers.

### Contact transmission

Donor animals were inoculated intranasally with 30 µL of rSeV-luc(M-F*) and were individually placed into cages containing 3 naïve contact mice at 24 h p.i. Bioluminescence was monitored daily until it remained consistently at background levels (∼15 days). Sera were collected on day 60 so that SeV-specific antibody levels could be measured as described above. On day 63, mice were challenged with 7000 PFU rSeV-luc(M-F*) administered i.n. and bioluminescence was measured daily.

## Supporting Information

Figure S1 Construction of luciferase-expressing Sendai viruses. (A) Nucleotide sequence of the firefly luciferase gene cassette. A pGEM3 cloning plasmid was engineered to contain flanking *Not*I restriction sites, the firefly luciferase reporter gene, gene end, and gene start sequences. (B) To insert the luciferase reporter gene cassette into three gene junctions, three pSeV genome plasmids were cloned to contain a unique *Not*I restriction site in each of the P-M, M-F, and F-HN gene junctions. For the pSeV-luc(M-F*) genome plasmid, the naturally occurring suboptimal start signal AGGGATAAAG was also mutated to the more efficient start signal AGGGTGAAAG to compensate for expected attenuation due to the addition of the foreign gene and additional gene junction. The firefly luciferase gene cassette (panel A) was subcloned from the pGEM3 plasmid into the pSeV genome plasmids using the *Not*I restriction sites. (C) Design of pSeV cDNA plasmids for the rescue of WT and recombinant Sendai viruses containing the luciferase reporter gene (luc). The locations of the Sendai virus genes nucleoprotein (N), polymerase (P), matrix (M), fusion (F), hemagglutinin-neuraminidase (HN), and large (L) protein are shown, as well as the T7 RNA polymerase promoter (T7) and hepatitis delta virus ribozyme sequence (ribo). Gene start sequences are shown in green and the naturally occurring, suboptimal AGGGATAAAG gene start sequence between the M and F genes of WT Sendai virus is shown in yellow. Gene end sequences are shown in red. The 3′ leader sequence upstream of the N gene and the 5′ trailer sequence downstream of the L gene are not shown for simplicity.
(TIF)

Figure S2 Sendai virus protein expression and incorporation into virions. (A) Sendai virus protein expression in LLC-MK2 cells. Confluent monolayers of LLC-MK2 cells were infected with recombinant Sendai viruses at an MOI of 5 PFU/cell and incubated for 16 h before radiolabeling with 50 µCi [^35^S]Promix (Amersham Pharmacia Biotech). Supernatant was incubated overnight at 4°C with mouse anti- NP, P, M, F, and HN monoclonal antibodies, and immune complexes were adsorbed on protein G-Sepharose (GE Healthcare), fractionated on 12% NuPAGE bis-Tris SDS-PAGE gels (Invitrogen), and visualized with a phosphorimager. (B) Ratios of Sendai virus protein expression. Protein expression was quantified with ImageQuant 5.2 software and normalized to the expression level of the N protein. The data represent the means (+/− standard deviation) from three experiments. (C) Sendai virus composition. Recombinant Sendai viruses were inoculated into 10-day-old embryonated chicken eggs. Allantoic fluid was harvested 72 h p.i. and centrifuged for 45 min at 3000 rpm to remove cellular debris. Supernatants were layered over a 60%–20% sucrose gradient and centrifuged at 24,000 rpm for 3.5 hrs to isolate virions. Isolated virions were diluted in TNE buffer and further purified by centrifugation over a 20% sucrose cushion at 24,000 rpm for 15 h. Virus pellets were resuspended in RIPA buffer and total protein concentrations were determined using the BCA protein assay kit (Thermo Sci.). Equal quantities of protein were separated on a 4%–12% SDS-PAGE gel, stained with Blue BANDit protein stain (Amresco), and dried in a BioRad gel dryer at 60°C for 45 minutes.
(TIF)

Figure S3 Immune responses of mice to infection with recombinant Sendai viruses. Groups of five 8-week-old 129/SvJ mice were intranasally inoculated with 30 µl containing 7,000 PFU of recombinant Sendai virus or PBS. On day 10 p.i., serum was collected and the mice were euthanized to recover bronchoalveolar lavage fluid (BALF). Experiments were performed twice with representative data shown. Each data point represents an individual animal and horizontal bars show group means. The numbers of CD4+ (A) and CD8+ (B) T cells recovered from BALF were determined by flow cytometry. (C) Luciferase-specific binding antibody titers in sera were determined by ELISA assays and are expressed as reciprocal endpoint dilutions. Firefly luciferase protein (Abcam) was used.
(TIF)

Figure S4 Bioluminescence and Sendai virus titers in the respiratory tracts of 129/SvJ mice. Groups of three 8-week-old mice were intranasally inoculated with 7,000 PFU of recombinant Sendai virus. (A) *In vivo* bioluminescence was measured for all three luciferase-expressing viruses on days 4 and 6 p.i., after which lungs were immediately harvested and homogenized so that *ex vivo* luciferase activity could be measured. A fit of the data with a least squares linear regression model yielded an *R* ^2^ value of 0.878. RLU denotes relative light units. (B) Comparison between light detected by the camera and viral titers of homogenates from the nasopharynx (triangles), trachea (circles), and lungs (squares). Each point represents data from a single mouse infected with rSeV-luc(M-F*) and studied on day 2, 3, 5, or 7 p.i.. Least squares linear regression yielded *R* ^2^ values of 0.864, 0.915 and 0.961 for the nasopharynx, trachea, and lungs, respectively.
(TIF)

Figure S5 Bioluminescence and viral titers in the respiratory tracts of BALB/c and 129/SvJ mice. Groups of three 8-week-old mice were intranasally inoculated with either 70 or 7,000 PFU of rSeV-luc(M-F*). (A) *In vivo* bioluminescence was measured in BALB/c mice infected with 7,000 PFU of virus on days 2, 3, 5, and 7 p.i., after which the animals were euthanized and tissues were harvested so that virus titers from tissue homogenates could be measured by plaque titration in LLC-MK2 cells. Correlations between virus titers in tissue homogenates and light detected by the camera were found with *R* ^2^ values of 0.928, 0.656, and 0.846 for the nasopharynx, trachea, and lungs, respectively. Virus titers in homogenates from the nasopharynx (B) and lungs (C) of both BALB/c- and 129/SvJ-strain mice infected with either 70 or 7,000 PFU of rSeV-luc(M-F*) were measured by plaque titration in LLC-MK2 cells. The data represent the mean virus titers of six mice (+/− standard deviation).
(TIF)

Figure S6 Tissue-specific timing and magnitude of Sendai virus spread in the respiratory tracts of intact mice after inoculation and contact transmission. In each group, one BALB/c or 129/SvJ mouse was inoculated intranasally with either 70 or 7,000 PFU of rSeV-luc(M-F*) and three contact animals were co-housed one day later as described in Figure 5. (A–D) Time until detection of bioluminescence in the nasopharynx (nasop.), trachea, and lungs (limit of detection: >6 log_10_ photons/s) after direct inoculation (open bars) and after contact transmission (solid bars). (E–H) Overall magnitude of infection after direct inoculation (open bars) and after contact transmission (solid bars) as determined by integration of daily measurements of total flux with respect to time using IgorPro software (Wavemetrics). The areas under the curve (AUC) of bioluminescence are expressed as the total amount of photons on a log_10_ scale. The experiment was performed in triplicate for 129/SvJ-strain mice (3 donor animals and 9 transmitted) and duplicate for BALB/c-strain mice (2 donor animals and 6 transmitted).
(TIF)
