# Supplementary figures and images for: Illumination of Parainfluenza Virus Infection and Transmission in Living Animals Reveals a Tissue-Specific Dichotomy
Source: PLoS Pathog. 2011 Jul 7;7(7):e1002134. doi: 10.1371/journal.ppat.1002134 (PMC3131265; doi:10.1371/journal.ppat.1002134)

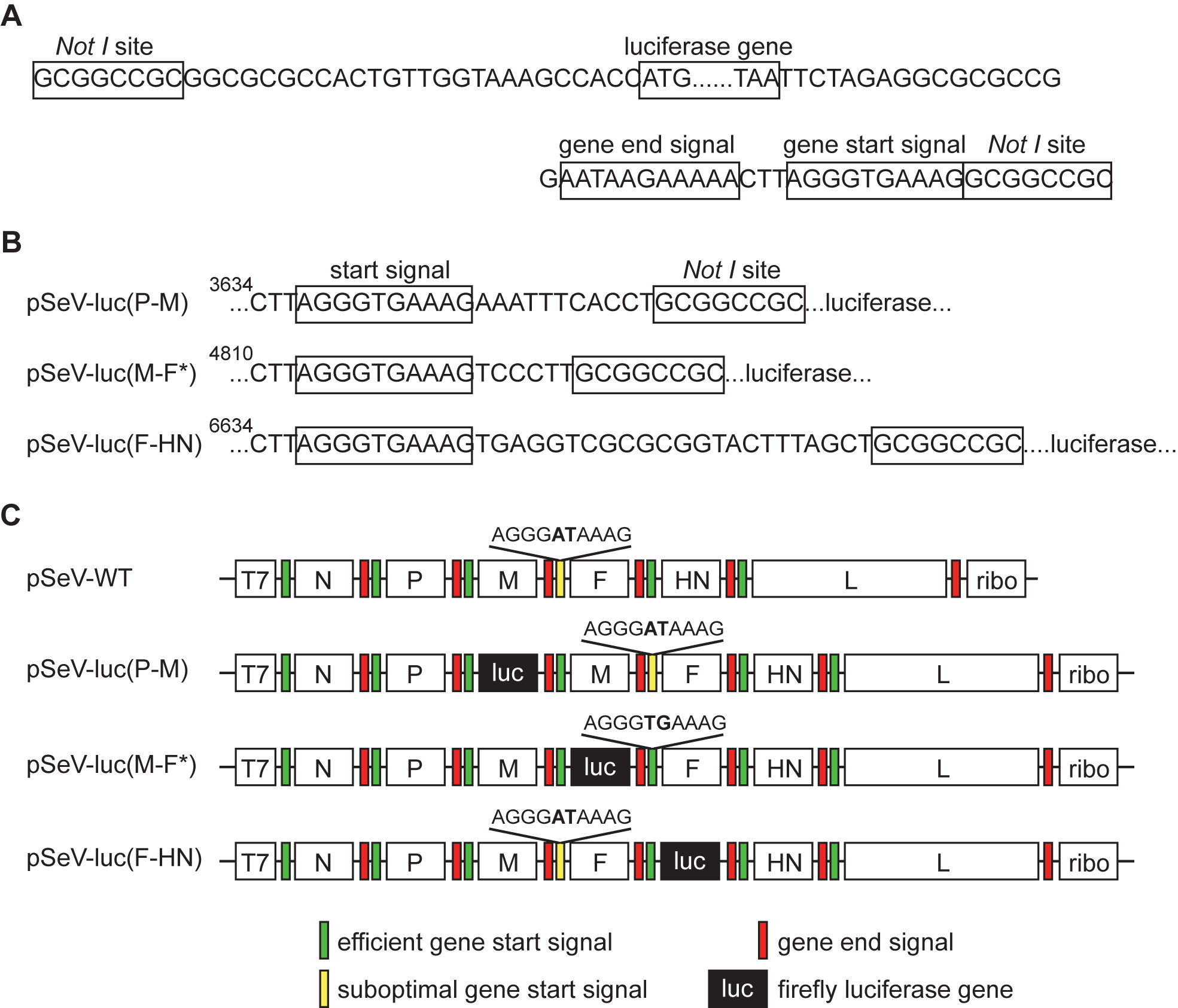

Supplement: Figure S1 — Construction of luciferase-expressing Sendai viruses. (A) Nucleotide sequence of the firefly luciferase gene cassette. A pGEM3 cloning plasmid was engineered to contain flanking NotI restriction sites, the firefly luciferase reporter gene, gene end, and gene start sequences. (B) To insert the luciferase reporter gene cassette into three gene junctions, three pSeV genome plasmids were cloned to contain a unique NotI restriction site in each of the P-M, M-F, and F-HN gene junctions. For the pSeV-luc(M-F*) genome plasmid, the naturally occurring suboptimal start signal AGGGATAAAG was also mutated to the more efficient start signal AGGGTGAAAG to compensate for expected attenuation due to the addition of the foreign gene and additional gene junction. The firefly luciferase gene cassette (panel A) was subcloned from the pGEM3 plasmid into the pSeV genome plasmids using the NotI restriction sites. (C) Design of pSeV cDNA plasmids for the rescue of WT and recombinant Sendai viruses containing the luciferase reporter gene (luc). The locations of the Sendai virus genes nucleoprotein (N), polymerase (P), matrix (M), fusion (F), hemagglutinin-neuraminidase (HN), and large (L) protein are shown, as well as the T7 RNA polymerase promoter (T7) and hepatitis delta virus ribozyme sequence (ribo). Gene start sequences are shown in green and the naturally occurring, suboptimal AGGGATAAAG gene start sequence between the M and F genes of WT Sendai virus is shown in yellow. Gene end sequences are shown in red. The 3′ leader sequence upstream of the N gene and the 5′ trailer sequence downstream of the L gene are not shown for simplicity. (TIF) [file ppat.1002134.s001.tif]

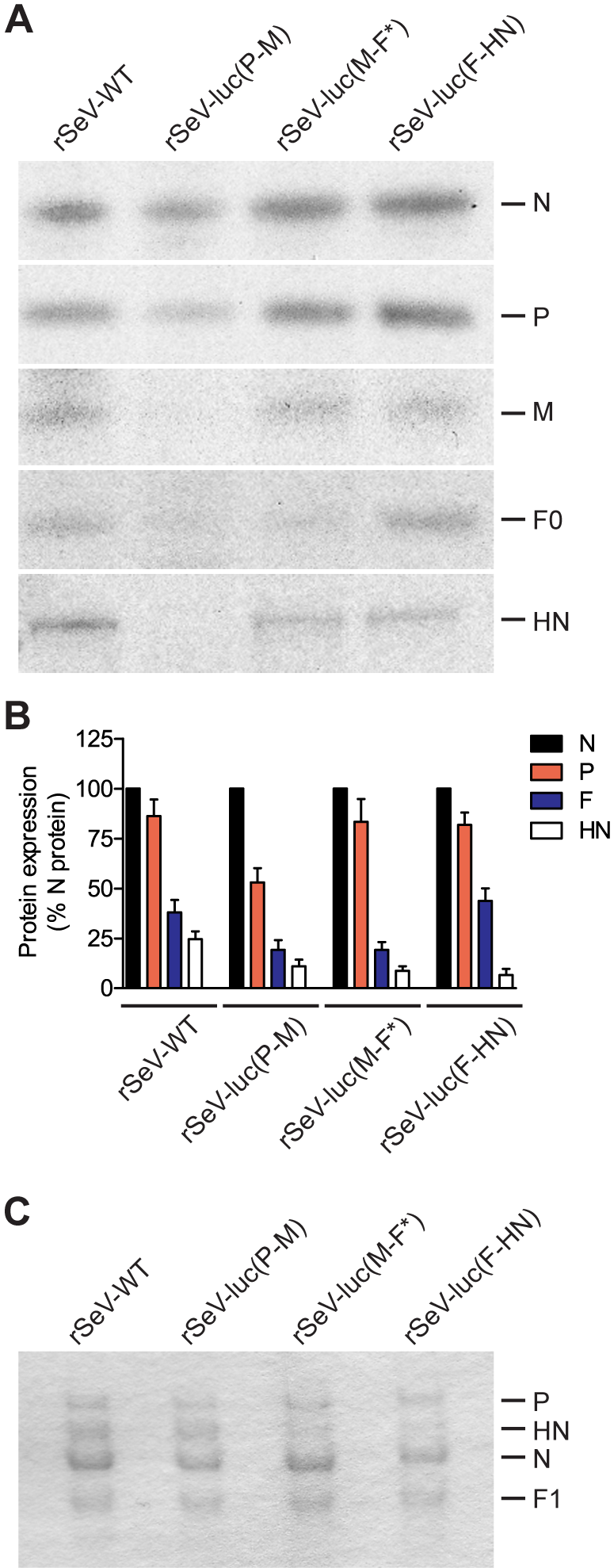

Supplement: Figure S2 — Sendai virus protein expression and incorporation into virions. (A) Sendai virus protein expression in LLC-MK2 cells. Confluent monolayers of LLC-MK2 cells were infected with recombinant Sendai viruses at an MOI of 5 PFU/cell and incubated for 16 h before radiolabeling with 50 µCi [35S]Promix (Amersham Pharmacia Biotech). Supernatant was incubated overnight at 4°C with mouse anti- NP, P, M, F, and HN monoclonal antibodies, and immune complexes were adsorbed on protein G-Sepharose (GE Healthcare), fractionated on 12% NuPAGE bis-Tris SDS-PAGE gels (Invitrogen), and visualized with a phosphorimager. (B) Ratios of Sendai virus protein expression. Protein expression was quantified with ImageQuant 5.2 software and normalized to the expression level of the N protein. The data represent the means (+/− standard deviation) from three experiments. (C) Sendai virus composition. Recombinant Sendai viruses were inoculated into 10-day-old embryonated chicken eggs. Allantoic fluid was harvested 72 h p.i. and centrifuged for 45 min at 3000 rpm to remove cellular debris. Supernatants were layered over a 60%–20% sucrose gradient and centrifuged at 24,000 rpm for 3.5 hrs to isolate virions. Isolated virions were diluted in TNE buffer and further purified by centrifugation over a 20% sucrose cushion at 24,000 rpm for 15 h. Virus pellets were resuspended in RIPA buffer and total protein concentrations were determined using the BCA protein assay kit (Thermo Sci.). Equal quantities of protein were separated on a 4%–12% SDS-PAGE gel, stained with Blue BANDit protein stain (Amresco), and dried in a BioRad gel dryer at 60°C for 45 minutes. (TIF) [file ppat.1002134.s002.tif]

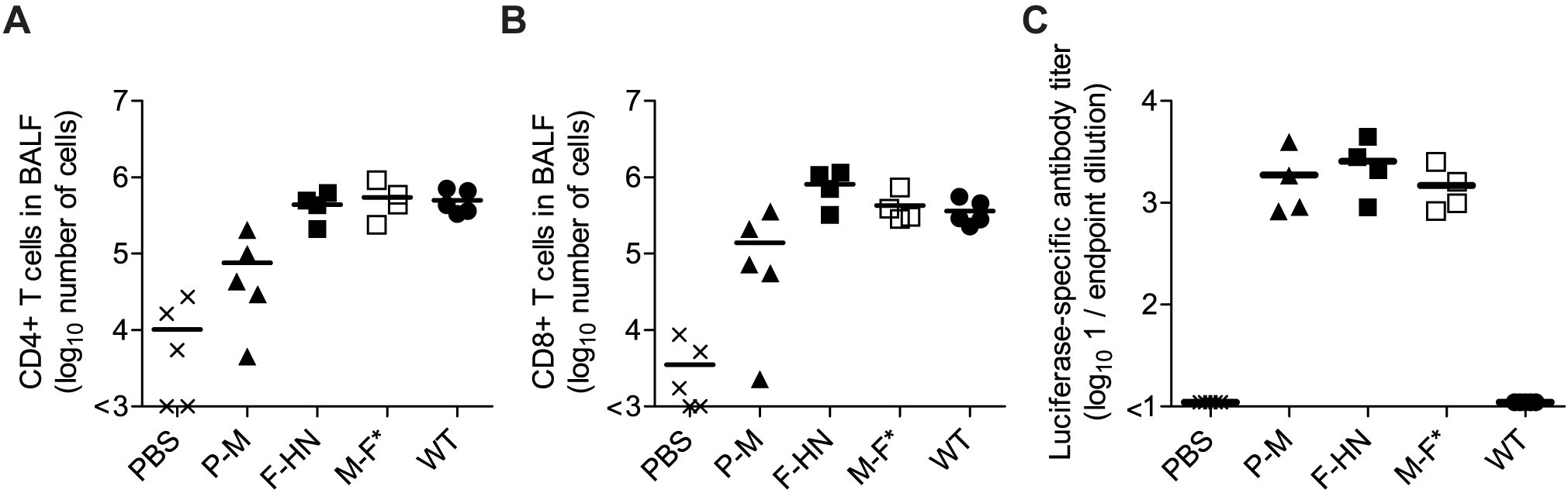

Supplement: Figure S3 — Immune responses of mice to infection with recombinant Sendai viruses. Groups of five 8-week-old 129/SvJ mice were intranasally inoculated with 30 µl containing 7,000 PFU of recombinant Sendai virus or PBS. On day 10 p.i., serum was collected and the mice were euthanized to recover bronchoalveolar lavage fluid (BALF). Experiments were performed twice with representative data shown. Each data point represents an individual animal and horizontal bars show group means. The numbers of CD4+ (A) and CD8+ (B) T cells recovered from BALF were determined by flow cytometry. (C) Luciferase-specific binding antibody titers in sera were determined by ELISA assays and are expressed as reciprocal endpoint dilutions. Firefly luciferase protein (Abcam) was used. (TIF) [file ppat.1002134.s003.tif]

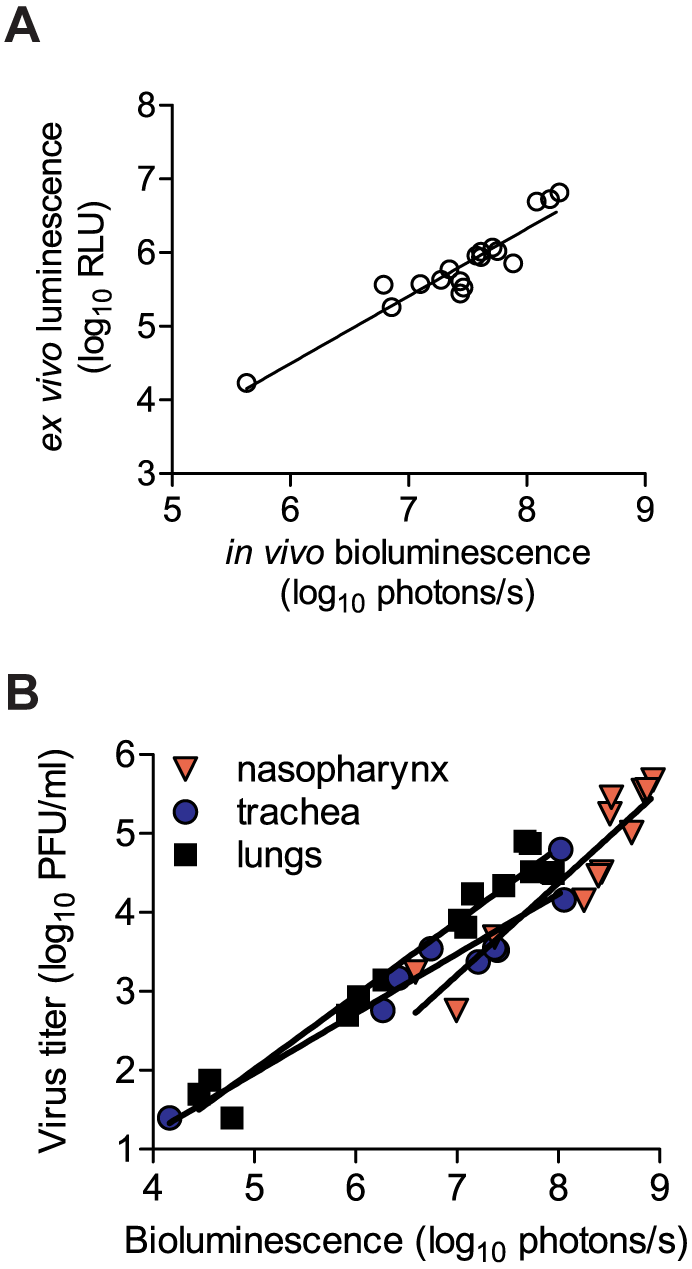

Supplement: Figure S4 — Bioluminescence and Sendai virus titers in the respiratory tracts of 129/SvJ mice. Groups of three 8-week-old mice were intranasally inoculated with 7,000 PFU of recombinant Sendai virus. (A) In vivo bioluminescence was measured for all three luciferase-expressing viruses on days 4 and 6 p.i., after which lungs were immediately harvested and homogenized so that ex vivo luciferase activity could be measured. A fit of the data with a least squares linear regression model yielded an R 2 value of 0.878. RLU denotes relative light units. (B) Comparison between light detected by the camera and viral titers of homogenates from the nasopharynx (triangles), trachea (circles), and lungs (squares). Each point represents data from a single mouse infected with rSeV-luc(M-F*) and studied on day 2, 3, 5, or 7 p.i.. Least squares linear regression yielded R 2 values of 0.864, 0.915 and 0.961 for the nasopharynx, trachea, and lungs, respectively. (TIF) [file ppat.1002134.s004.tif]

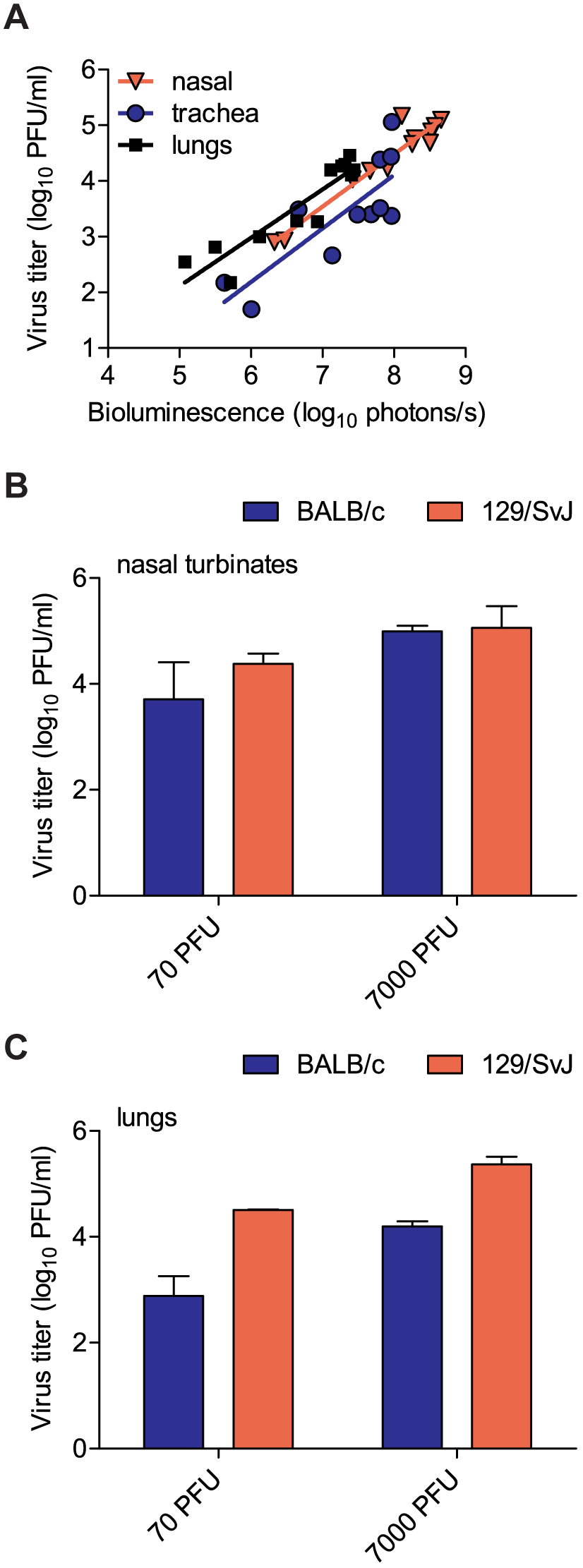

Supplement: Figure S5 — Bioluminescence and viral titers in the respiratory tracts of BALB/c and 129/SvJ mice. Groups of three 8-week-old mice were intranasally inoculated with either 70 or 7,000 PFU of rSeV-luc(M-F*). (A) In vivo bioluminescence was measured in BALB/c mice infected with 7,000 PFU of virus on days 2, 3, 5, and 7 p.i., after which the animals were euthanized and tissues were harvested so that virus titers from tissue homogenates could be measured by plaque titration in LLC-MK2 cells. Correlations between virus titers in tissue homogenates and light detected by the camera were found with R 2 values of 0.928, 0.656, and 0.846 for the nasopharynx, trachea, and lungs, respectively. Virus titers in homogenates from the nasopharynx (B) and lungs (C) of both BALB/c- and 129/SvJ-strain mice infected with either 70 or 7,000 PFU of rSeV-luc(M-F*) were measured by plaque titration in LLC-MK2 cells. The data represent the mean virus titers of six mice (+/− standard deviation). (TIF) [file ppat.1002134.s005.tif]

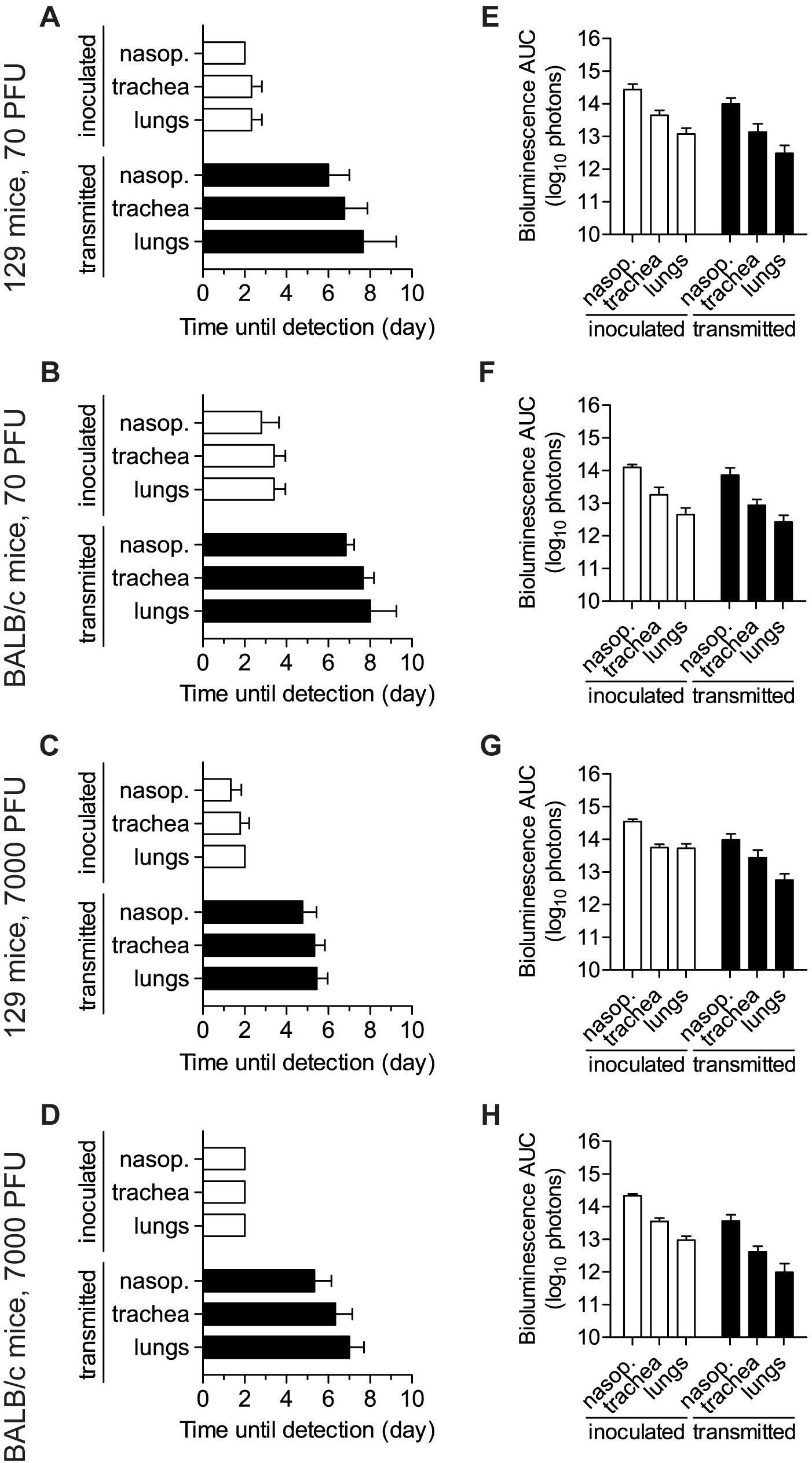

Supplement: Figure S6 — Tissue-specific timing and magnitude of Sendai virus spread in the respiratory tracts of intact mice after inoculation and contact transmission. In each group, one BALB/c or 129/SvJ mouse was inoculated intranasally with either 70 or 7,000 PFU of rSeV-luc(M-F*) and three contact animals were co-housed one day later as described in Figure 5. (A–D) Time until detection of bioluminescence in the nasopharynx (nasop.), trachea, and lungs (limit of detection: >6 log10 photons/s) after direct inoculation (open bars) and after contact transmission (solid bars). (E–H) Overall magnitude of infection after direct inoculation (open bars) and after contact transmission (solid bars) as determined by integration of daily measurements of total flux with respect to time using IgorPro software (Wavemetrics). The areas under the curve (AUC) of bioluminescence are expressed as the total amount of photons on a log10 scale. The experiment was performed in triplicate for 129/SvJ-strain mice (3 donor animals and 9 transmitted) and duplicate for BALB/c-strain mice (2 donor animals and 6 transmitted). (TIF) [file ppat.1002134.s006.tif]
